# Psychometric properties of a COVID-19 health literacy scale in a sample of German school principals applying Rasch analysis

**DOI:** 10.1186/s12889-024-20648-w

**Published:** 2024-11-11

**Authors:** Marlene Meyer, Kevin Dadaczynski, Melanie Messer, Orkan Okan

**Affiliations:** 1 WHO Collaborating Centre for Health Literacy, TUM Health Literacy Unit, Department of Health and Sport Sciences, TUM School of Medicine and Health, Technical University of Munich, Munich, Germany; 2 Public Health Centre Fulda, Fulda University of Applied Sciences, Fulda, Germany; 3 Centre for Applied Health Science, Leuphana University Lueneburg, Lueneburg, Germany; 4 Institute of Nursing Science, University Hospital Würzburg, Würzburg, Germany; 5 Department of Nursing Science, University of Würzburg, Würzburg, Germany

**Keywords:** COVID-19 health literacy, Rasch analysis, Psychometric properties, HLS-COVID-Q22, German school principals

## Abstract

**Background:**

During the COVID-19 pandemic, health literacy was found to be an asset to manage health-related information. The HLS-COVID-Q22 has been developed to measure COVID-19 health literacy. External validation needs to be assessed in different populations to verify the questionnaire’s functioning. The present study aimed to evaluate the psychometric properties of the HLS-COVID-Q22 in a sample of German school principals.

**Methods:**

The sample consisted of 2187 German school principals who completed the HLS-COVID-Q22 online from April to March 2021. The data was analyzed using Rasch analysis, applying the Partial Credit Model for polytomous data. Dimensionality, item fit statistics and rating scale functioning was tested. Values for item difficulty and person ability as well as reliability indices were computed.

**Results:**

Unidimensionality could be confirmed. The rating scale categories worked as intended, participants used every rating step category. Generally, item fit was verified. One item showed potential misfit but could remain in the questionnaire as excluding the item did not reduce reliability. A person separation index of 3.41 and person reliability of 0.92 showed excellent differentiation between COVID-19 health literacy levels. Furthermore, the values for item separation of 20.08 and item reliability of 1.0 indicate good construct validity.

**Conclusions:**

The German version of the HLS-COVID-Q22 appears to be a reliable measurement tool for the target population. Evidence for construct, statistical and fit validity was collected. Future studies need to test additional types of validity like convergent and divergent validity to further evaluate the questionnaire. Moreover, the psychometric properties of the translated versions of the HLS-COVID-Q22 should be compared using Rasch analysis.

## Background

With the COVID-19 outbreak, there has been a rapid increase in health information concerning the virus [1, 2]. This led to an epidemic of information, known as *infodemic*, which is characterized by a vast spread of not only correct but false information via diverse communication technologies in people’s information ecosystems [3]. The World Health Organization (WHO) classifies infodemics as a threat to public health [4, 5]. For instance, people might get overloaded and confused by the sheer amount of information that was suddenly available and struggle to differentiate between reliable information and false information. In this context, health literacy has been highlighted as a personal resource for managing health information and navigating the information ecosphere [6, 7]. Health literacy (HL) can be defined as the ability to access, understand, appraise and apply health information to reach health-related decisions [8]. Accordingly, COVID-19 HL is the ability to access, understand, appraise and apply health information to reach health-related decisions in the context of the COVID-19 pandemic. In a cross-sectional study, HL has been associated with mental health during the pandemic and adherence to COVID-19 preventive measures [9].

The HLS-COVID-Q22 questionnaire was developed by Okan et al. to measure COVID-19 HL, based on the European Health Literacy Survey Questionnaire (HLS-EU-Q47), which is a widely used instrument in health literacy research [10]. The HLS-EU-Q47 measures a comprehensive concept of health literacy in adult populations and has been validated using principal component analysis (PCA) [11, 12], confirmatory factor analysis (CFA) [13, 14], and Rasch modelling [15]. Several reliable, validated short forms exist, such as the HLS-Q12 [16]. Therefore, the HLS-COVID-Q22 was adapted to the context of the COVID-19 pandemic for a general adult population survey [10].

Classical test theory (CTT) or item response theory (IRT) can be used to validate a questionnaire [17]. Whereas CTT is predominantly used to evaluate the psychometric properties of surveys with an interval variable, IRT is used for surveys with a categorical variable. The Rasch model is often classified as 1-parameter IRT model. However, although the IRT models and the Rasch model seem similar, there is a philosophical difference [18]. IRT models are allowed to be modified to fit data sets, whereas Rasch models are not [19]. According to Thurstone, a measurement tool has to be independent of what it measures, in particular, independent of the sampling or else, validity of the tool is impaired [20]. Compared to IRT models, Rasch models will not be altered to fit the underlying data set, the data set is examined to fit the Rasch model [21]. Therefore, Boone et al. argue that the Rasch model is a measurement tool as defined by Thurstone [18].

Currently, the HLS-COVID-Q22 has been used and validated, using CTT, in some studies with different target groups [10, 22, 23]. However, several comprehensive validation studies are needed to ensure the feasibility of a novel measurement tool for various populations [24]. Additionally, the HLS-COVID-Q22 has not yet been validated by applying Rasch analysis, even though Rasch analysis is already a well-established validation method used in other health literacy questionnaires [15, 16, 25, 26]. One advantage in choosing Rasch analysis instead of CFA lies in the categorical nature of the items of the HLS-COVID-Q22. A prerequisite of using the standard continuous CFA model is a continuous variable [27]. When a categorical variable is treated as continuous, biased parameter estimates and model test statistics might occur, especially with a small number of categories [28]. Using an adjusted estimation method can mitigate these effects, e.g., robust categorical least squares [27]. However, Rasch analysis transforms the categorical responses of the HLS-COVID-Q22 into linear measures [18]. Since the HLS-COVID-Q22 has only 4 response categories, Rasch analysis might be better suited for validation purposes. Furthermore, the German version of the HLS-COVID-Q22 has been tested with CFA for construct validity and Cronbach’s Alpha as indicator for reliability in a representative sample of German internet users [10]. However, more than one type of validity and reliability is needed for a comprehensive psychometric evaluation of a measurement tool [29, 30]. Rasch analysis provides several different indicators to assess the validity and reliability of a measurement tool, e.g., more than one reliability index, item fit statistics and methods to evaluate the rating scale [18, 29].

The COVID-19 pandemic negatively affected the education sector, e.g., school closures, the change from face-to-face to online teaching and the implementation of hygiene regulations [31–33]. This led to a detrimental impact on mental health, teaching and learning, quality of life, and physical health in school populations (students, teachers, parents, school administration) [31]. School principals are proposed to be the gatekeepers for the implementation of school health promotion and prevention [34]. School principals’ behavior is associated with student achievement, teacher well-being and health promoting school activities [35]. However, research on school principals’ role in health promotion tends to be neglected and evidence on their HL is scarce [34, 36]. Recently, male school principals’ HL levels were associated with the implementation of health promotion in schools [36]. In this context, COVID-19 HL might not only be a personal resource for school principals’ own health, but also an influencing factor for COVID-19 related health promotion in the whole school community. School principals’ ability to manage COVID-19 related health information (e.g., trusting and applying reliable information while rejecting misinformation) is proposed to be essential for making informed decisions concerning the health of the whole school environment [37, 38].

The present study aims to evaluate the psychometric properties of the German version of the HLS-COVID-Q22 in a sample of school principals from the COVID-19 Health Literacy School Principals Survey (COVID-HL school survey) [39]. In particular, applying Rasch analysis, the goals were to (1) transform categorical responses of the HLS-COVID-Q22 into linear measures, (2) test for unidimensionality, (3) compute values for item difficulty (item measure) and person ability (person measure) on one dimension and provide several indices to assess the quality of the measurement tool, e.g. (4) reliability, (5) item fit statistics as well as rating scale functioning [18, 29].

## Methods

### Study design and study population

The Covid-19 Health Literacy Network (COVID-HL Network; now renamed the Global Health Literacy Research Network, GLOBHL) was established in March 2020 [40, 41]. The network’s research focuses on different aspects of health literacy, digital health issues, and how people manage and use health information. One of the network’s goals was to initiate international collaborative studies to enable the generation of national data on health literacy during the COVID-19 pandemic and to facilitate cross-country comparisons. The COVID-HL school survey was conducted in about 20 countries [42]. In Germany, the online-based study was conducted from the 9th of March to 13th of April 2021 during the third wave of infection [40]. The study population included school principals, deputies, and members of the school management team from four German federal states: Baden-Wuerttemberg, Hesse, Lower Saxony, and North Rhine-Westphalia. Inclusion criteria were that participants had to be school principals or part of the school management team. However, people not belonging to the target population were excluded from the data set after data collection has finished. In cooperation with the school principals’ associations of these federal states, school principals received an invitation to participate in the survey through e-mail distribution lists. After about 10 days, an e-mail reminder was send to increase the participation rate. Participants gave written informed consent prior to taking part in the survey. They were informed about the purpose of the study, that participation is voluntary and anonymous and that they could end their participation in the survey at any time. This study was approved by the Bielefeld University Ethics Board (Reference No 2021-030).

Overall, 2187 participants took part in the German survey. 89 participants were excluded from data analysis due to not having answered the HLS-COVID-Q22 instrument (83 participants) and not being a school principal, a deputy or part of the school management team (6 participants). 66.1% of the participants were female, 33.8% male, and 0.1% did not specify. The age ranged from 27 to 68 years with a mean of *M* = 51 years. More than half (53%) worked at a primary school, whereas 47% worked at a secondary school. 84.2% were school principals and 15.8% were deputies or a school management team member. 2031 participants were included in the final data analysis. 67 “extreme participants” were excluded due to having achieved the maximal possible raw score on the questionnaire. For those participants, the real participant ability is not measurable since it could be exactly at the maximal possible raw score of the questionnaire or in an unquantifiable amount above [18]. Accordingly, participants with the minimum possible raw score on the questionnaire would have been excluded if there had been any in the data set. Hence, excluding those respondents is justifiable based on an infinite measurement error that might affect the validation of a measurement tool.

### Measurement tool

The development of the HLS-COVID-Q22 and item descriptions have been reported by Okan et al. [10]. The instrument assesses the COVID-19 health literacy of adults. It consists of 22 items and four subscales: accessing (6 items), understanding (6 items), appraising (5 items) and applying (5 items) health-related information in the context of the COVID-19 pandemic. Participants were asked to rate on a 4-point likert-type scale (“very difficult”, “difficult”, “easy”, “very easy”), how easy or difficult they perceive the items, for example item 1: “On a scale from very easy to very difficult, how easy would you say it is to find information about the coronavirus on the internet?” [10].

### Data analyses

#### Rasch analysis

An initial Rasch analysis has been conducted to examine data quality as a requirement for using the Rasch model [18]. Different fit indices describe how well the data conform to the Rasch model. Since the Rasch model is seen as a measurement tool by definition of Thurstone, a misfit of data and model expectations needed to be investigated, shedding light on the differences of the theory used to design the questionnaire and participants’ patterns of answer [18]. If the data do not fit the Rasch model, the questionnaire might not measure the underlying concept (COVID-19 HL) very well or the theoretical assumptions of the concept of COVID-19 HL might be flawed. Because the HLS-COVID-Q22 is a likert-type rating scale, consisting of subscales, the data set was analyzed using WINSTEPS^®^ software, applying the Partial Credit Model for polytomous data [43]. WINSTEPS^®^ uses the two estimation processes PROX (Normal Approximation Estimation Algorithm) and JMLE (Joint Maximum Likelihood Estimation) to compute person measures and item measures [44]. Through Rasch analysis, participant’s responses to categorical items were computed into one interval scaled linear person measure, reflecting the participant’s ability [18]. Furthermore, for each item, an item measure was computed, reflecting the difficulty of that item. Both, person measures and item measures share the same unit (logits). They are displayed on the same continuum of the measured dimension (COVID-19 HL), allowing to compare persons via person measures and person abilities in relation to item difficulties. Rasch analysis allows to compute reliable person and item measures even if not every item has been answered. In the present questionnaire, there were 1.3% of missing data.

#### Dimensionality

A sum score of the HLS-EU-Q47 was introduced and adopted for the HLS-COVID-Q22 [10], implying that the four subscales accessing, understanding, appraising and applying define one dimension, COVID-19 HL. Therefore, unidimensionality of the measurement tool was tested as a prerequisite of the Rasch model. Principal component analysis of the residuals (PCAR) analysis has been conducted to test for unidimensionality [29]. The unexplained part of the data, the residuals, should be random noise. If the Eigenvalue is above 2.0, it could indicate a second dimension [29]. Because multidimensionality could not be ruled out with PCAR, disattenuated correlation between person measures of one set of items (possible dimension 1) and another set of items (possible dimension 2) were also investigated. A value of 0.7 is currently used as a cut off to identify a correlation as sufficient, indicating that both item sets measure the same dimension [29].

Additionally, local dependence of items was tested. Two items are locally dependent when the responses to one item are statistically dependent on the responses of another item [44]. This might occur if the items are redundant or they incorporate another dimension. According to Linacre, correlations of the residuals of the items of 0.40 show low dependency and are concerning above 0.70 [44].

#### Item fit

To investigate item fit, Item Outfit MNSQ will be reported as they are sensitive to outliers [18, 44]. An item misfits, for instance, if participants with high person measures (high level of COVID-19 health literacy) respond to an easy item unexpectedly with lower rating scale categories instead of higher rating categories. Additionally, Item Infit MNSQ provides an index for responses near a particular item difficulty [18, 44] . Here, an item could show a misfit, if participants respond to that item unexpectedly differently (e.g., choosing category “very easy”) than to other items with similar item difficulty (e.g., choosing category “very difficult”). Reasonable mean-square fit statistic values should be between 0.6 and 1.4 for rating scale surveys, according to Wright and Linacre [45]. When the data perfectly fit the model, Infit and Outfit MNSQ values of 1 would be expected [18, 44]. If an item showed MNSQ values outside of the range (0.6–1.4), standardized Z values (ZSTD) were examined. An item outside of the MNSQ value range and with ZSTD values of ± 2 or higher should be further investigated [18]. A misfitting item was removed, another Rasch analysis without the item was conducted and reliability indices were compared to the initial analysis to assess changes in quality of the measurement tool. Furthermore, mean-square fit statistics were computed for person measures but only reported as mean summary statistics to examine sample fit.

In addition to item fit, item discrimination was evaluated to assess item performance. Item discrimination reflects how well an item discriminates between participants with low person measures compared to participants with high person measures [29]. For every item, a gap has been computed between participants who selected the lowest rating step category (“very difficult”) and respondents who selected the highest rating step category (“very easy”). Item discrimination is as higher as larger the computed gap.

Differential item functioning (DIF) analysis was conducted to examine if the items performed differently for specific subgroups [18]. DIF analysis was performed for age and sex (female vs. male). For the age analysis, the data were split by mean age (< 51 vs. > 51 years). An item was investigated as showing potential DIF, with an Alpha value of *p* < .05. Then an evaluation of effect size was conducted using a suggested threshold of 0.64 for DIF contrast, implying moderate to large DIF [18, 44].

#### Reliability

Four indices were computed to evaluate reliability of the measurement tool: person reliability, item reliability, person separation and item separation [18, 44]. Person reliability reflects how reliable the person measures are, while item reliability reflects how reliable the item measures are. Person and item reliability range from 0 to 1 and reliability values of > 0.90 are needed to differentiate between 4 levels of health literacy. Person separation is a metric that indicates how well the measurement tool differentiates between participants’ ability levels. Item separation is an indicator of construct validity. Person separation and item separation can range from 0 to infinity, avoiding any ceiling effect. Person separation of 1.5 is termed as acceptable, 2.0 as good and 3.0 as excellent [46–48]. Item separation of 2.5 is needed for an analysis of group level [49].

#### Rating scale

It was examined how well the rating scale functioned. In the first step, it was determined if every category (“very difficult”, “difficult”, “easy”, “very easy”) was used by participants [18]. If there are too many categories and respondents are not using every category that may detriment the measurement tool as people lose time thinking about every additional rating step, and there might be a misconception about the dimension being measured. Therefore, a rating scale should have as many categories as required to differentiate between participants’ ability levels. The probability of choosing a category was assessed, and it was checked whether each category was most likely for specific person measure-item difficulty combinations.

In the next step, rating step ordering has been examined for every item [18]. The mean person measure of all participants who chose this category was investigated for every category of every item. There should be an increase in the mean person measure for every increase in rating step category, that is, the mean person measure of “very difficult” should be the lowest, of “difficult” the second lowest, of “easy” the second highest and of “very easy” the highest. Items with rating step disordering were further investigated.

## Results

### Dimensionality

The variance explained by Rasch measures was 52.1%, with 38.4% of variance explained by person measures and 13.7% by item measures. The unexplained variance amounted to 47.9%. Principal component analysis of the residuals resulted in an Eigenvalue of the unexplained variance in the first contrast of 2.39. Since an Eigenvalue of 2 is used as a cut-off for possible multidimensionality, further investigation was carried out. When comparing contrast 1 loadings (Table 1), it is apparent that items of the subscales “appraising” and “applying” show positive loadings. In contrast, the items of the subscales “accessing” and “understanding” show negative loadings, except for two items of the subscale “accessing”. Accordingly, one Rasch analysis was conducted only including items of the subscales “accessing” and “understanding” and a second Rasch analysis including items of the subscales “appraising” and “applying”. A disattenuated correlation analysis was then calculated between person measures of those two sets of items. The cut-off of 0.70 has been exceeded with a disattenuation correlation value of 0.866, suggesting that both item sets measure the same dimension, strengthening the assumption of unidimensionality of the HLS-COVID-Q22.

**Table 1:** Contrast 1 loadings from principal component analysis

Loading	Item number	Subscale	Loading	Item number	Subscale
0.52	16	Appraising	− 0.52	12	Understanding
0.47	17	Appraising	− 0.49	2	Accessing
0.36	20	Applying	− 0.49	11	Understanding
0.32	6	Accessing	− 0.48	3	Accessing
0.32	19	Applying	− 0.43	10	Understanding
0.30	22	Applying	− 0.40	1	Accessing
0.18	15	Appraising	− 0.23	9	Understanding
0.12	14	Appraising	− 0.15	4	Accessing
0.12	21	Applying	− 0.06	8	Understanding
0.07	5	Accessing	− 0.02	7	Understanding
0.05	18	Applying			
0.02	13	Appraising			

Standardized residual loadings for items are sorted by loading. Item classification by subscale is visualized for positive and negative loadings

In Table 2, the largest standardized residual correlations to identify locally dependent items are displayed (WINSTEPS^®^ Output Table 23.99). Three item-pairs showed low dependency with standardized residual correlations above 0.40: 0.42 between Items 1 and 2, 0.45 between Items 19 and 20, 0.60 between Items 11 and 12. No concerning local dependence (correlation > 0.70) of items was detected.

**Table 2:** Largest standardized residual correlations of items

Correlation	Item 1	Subscale	Item 2	Subscale
0.60	11	Understanding	12	Understanding
0.45	19	Applying	20	Applying
0.42	1	Accessing	2	Accessing
0.38	14	Appraising	15	Appraising
0.35	6	Accessing	16	Appraising
0.32	2	Accessing	3	Accessing
0.22	9	Understanding	10	Understanding
0.19	16	Appraising	17	Appraising
− 0.22	2	Accessing	17	Appraising
− 0.21	10	Understanding	16	Appraising
− 0.21	2	Accessing	16	Appraising
− 0.21	10	Understanding	17	Appraising
− 0.20	6	Accessing	18	Applying
− 0.19	6	Accessing	12	Understanding
− 0.19	7	Understanding	13	Appraising
− 0.19	12	Understanding	17	Understanding
− 0.18	1	Accessing	17	Understanding
− 0.18	3	Accessing	16	Understanding
− 0.18	12	Understanding	16	Understanding
− 0.18	13	Appraising	19	Applying

Standardized residual correlations of two items are sorted by loading. Item classification by subscale is visualized for positive and negative loadings

### Item fit

Table 3 shows the summary statistics for items and persons. The mean item measure is standardized to 0.00 logits. The mean person measure is 2.07 logits, indicating that people frequently responded agreeable to the items. General item fit is underlined by mean item Infit MNSQ of 0.99 and mean item Outfit MNSQ of 0.99. Furthermore, mean person Infit MNSQ of 1.03 and mean person Outfit MNSQ of 0.99 indicate that the sample generally fit.

**Table 3:** Summary statistics

	Mean Measure	Model S.E.	Mean Infit MNSQ	Mean Outfit MNSQ
**Item**	0.00	0.04	0.99	0.99
**Person**	2.07	0.45	1.03	0.99

S.E. = standard error; MNSQ = mean square standardized residual

Item statistics are displayed in Table 4. Item measures range from − 1.49 to 1.69. Item 8 shows potential misfit with an Infit MNSQ value of 1.55 (Infit ZSTD = 9.90) and Outfit MNSQ value of 1.68 (Outfit ZSTD = 9.90), exceeding the upper cut-off value of 1.40 for reasonable mean-square fit statistic and the cut-off value of ZSTD > 2.00. The remaining 21 items show adequate Infit MNSQ values, ranging from 0.72 to 1.37, and Outfit MNSQ values, ranging from 0.67 to 1.36. Mean item discrimination was 4.06 logits. Item 3 showed the lowest discrimination with a gap of 2.76 logits. Additionally, items 9, 22 and 8 displayed rather low discrimination with gaps of 3.09, 3.40 and 3.49, respectively. Item 15 revealed the highest discrimination with a gap of 5.09 logits. Also, items 21 and 10 showed particularly high discrimination, with gaps of 4.99 and 4.74. Closest to the mean item discrimination were Items 12, 5 and 6, with gaps of 3.93, 3.94 and 4.28.

**Table 4:** Item statistics

Item number	Item measure	Model S.E.	Infit MNSQ	Infit ZSTD	Outfit MNSQ	Outfit ZSTD	Discrimination gap	Subscale
8	1.52	0.04	1.55	9.90	1.68	9.90	3.49	Understanding
22	− 0.22	0.05	1.37	9.42	1.36	7.58	3.40	Applying
3	− 0.39	0.04	1.14	3.99	1.36	6.62	2.76	Accessing
13	1.99	0.04	1.33	9.75	1.34	9.90	4.36	Appraising
17	1.69	0.04	1.19	5.99	1.22	6.73	4.61	Appraising
5	− 0.11	0.04	1.17	4.69	1.21	4.36	3.94	Accessing
9	− 0.22	0.05	1.12	3.39	1.19	4.18	3.09	Understanding
16	1.11	0.04	1.14	4.38	1.14	4.20	4.50	Appraising
6	1.05	0.04	1.05	1.66	1.08	2.27	4.28	Accessing
4	− 0.40	0.04	0.94	-1.90	1.00	− 0.05	3.79	Accessing
1	-1.49	0.05	0.95	-1.49	0.96	− 0.43	3.69	Accessing
14	− 0.20	0.04	0.89	-3.35	0.87	-3.20	4.47	Appraising
21	0.83	0.04	0.87	-4.22	0.85	-4.39	4.99	Applying
2	-1.15	0.05	0.86	-4.36	0.86	-2.14	3.46	Accessing
19	− 0.65	0.05	0.83	-5.22	0.76	-5.43	3.63	Applying
20	− 0.36	0.05	0.81	-5.65	0.73	-6.59	4.47	Applying
12	− 0.27	0.05	0.78	-6.70	0.73	-6.72	3.93	Understanding
7	-1.22	0.05	0.77	-7.53	0.70	-5.54	3.62	Understanding
11	− 0.31	0.05	0.76	-7.65	0.69	-8.00	4.38	Understanding
15	− 0.57	0.04	0.73	-8.87	0.67	-8.00	5.09	Appraising
18	0.03	0.05	0.73	-8.39	0.68	-9.00	4.57	Applying
10	− 0.66	0.05	0.72	-8.90	0.67	-7.73	4.74	Understanding

Item statistics are shown in fit order. S.E. = standard error; MNSQ = mean square standardized residual; ZSTD = standardized Z value

The results of the DIF analysis are shown in Table 5. No DIF was detected for age or sex.

**Table 5:** Differential item functioning for age and sex

Item	< 51 years vs. > 51 years		Female vs. Male	
	DIF contrast	Probability	DIF contrast	Probability
1	− 0.21	0.03	0.03	0.80
2	− 0.22	0.02	0.17	0.09
3	− 0.10	0.25	0.21	0.03
4	0.07	0.45	0.00	1.00
5	0.12	0.18	− 0.13	0.17
6	0.18	0.02	− 0.09	0.28
7	0.04	0.66	0.14	0.17
8	0.20	0.01	0.57	0.00
9	0.00	1.00	0.02	0.82
10	− 0.13	0.17	0.00	1.00
11	− 0.09	0.36	0.00	1.00
12	0.00	1.00	0.00	1.00
13	0.00	1.00	− 0.63	0.00
14	− 0.02	0.81	− 0.21	0.03
15	0.00	1.00	0.07	0.43
16	0.17	0.04	− 0.19	0.03
17	0.00	1.00	− 0.21	0.02
18	− 0.13	0.15	0.23	0.02
19	− 0.03	0.78	0.14	0.17
20	− 0.09	0.37	0.06	0.54
21	− 0.14	0.10	0.03	0.77
22	0.25	0.01	− 0.08	0.42

### Reliability

In Table 6, reliability indices are shown. The initial Rasch analysis computed values for person separation of 3.41 and person reliability of 0.92, indicating that the HLS-COVID-Q22 differentiates excellent between participants 4 COVID-19 health literacy levels. Additionally, the values for item separation of 20.08 and item reliability of 1.0 indicate good construct validity of the HLS-COVID-Q22 and justify analysis at the group level.

**Table 6:** Reliability indices

	Item		Person	
	With	Without	With	Without
**Separation**	20.08	19.33	3.41	3.34
**Reliability**	1.0	1.0	0.92	0.92

Item and person separation and reliability values are contrasted when Item 8 is included (with) and excluded (without)

Item fit analysis revealed a potential misfit of Item 8 (“On a scale from very easy to very difficult, how easy would you say it is to understand recommendations of authorities regarding protective measures against coronavirus infection?” [10]). Therefore, another Rasch analysis was conducted, excluding Item 8. This analysis computed values for person separation of 3.34 and person reliability of 0.92 and values for item separation of 19.33 and item reliability of 1.0. When comparing the reliability indices of both analyses, excluding Item 8 results in slightly decreased reliability of both person separation and item separation. Thus, excluding Item 8 from the HLS-COVID-Q22 does not increase the reliability of the measurement tool.

### Rating scale

**Fig. 1:**
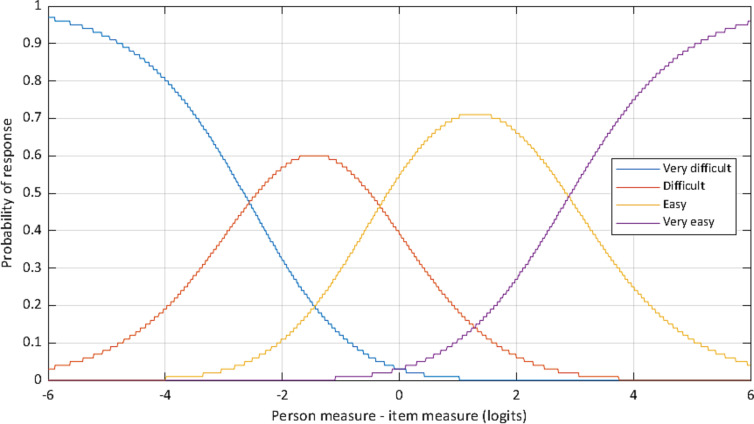
Probability curves of rating scale categories. Figure 1 shows the probability of a particular category being selected as a function of person measure and item difficulty. Blue curve = very difficult, orange curve = difficult, yellow curve = easy, purple curve = very easy

For all subscales (accessing, understanding, appraising, applying), participants used every rating step category (“very difficult”, “difficult”, “easy”, “very easy”). Every category is most probable for specific person measure-item difficulty combinations. As an example, probability curves of the four rating step categories as a function of person measure and item difficulty are shown for the subscale accessing in Fig. 1. Here, every curve (blue curve = “very difficult”, orange curve = “difficult”, yellow curve = “easy”, purple curve = “very easy”) shows the highest probability of being chosen for some sections along the X-axis. For example, selecting “easy” (yellow curve) would be predicted for a participant if the person measure of that participant is 1 logit higher than the item measure of the item in question.

Step ordering is displayed in Table 7. Here, Item 1 is presented as an example for step ordering while Item 2 is presented as an example for step disordering. Generally, there has been an increase in the mean person measure for every increase in rating step category. However, Items 2, 3, 7, 9 and 12 showed step disordering. All of those five items showed no increase but a decrease in mean person measure from rating category 1 (“very difficult”) to rating category 2 (“difficult”).

**Table 7:** Step ordering of the rating scale

	Item 1			Item 2		
Rating scale category	Count	Percent	Mean ability	Count	Percent	Mean ability
Very difficult	5	0	− 0.68	9	0	− 0.23
Difficult	60	3	− 0.32	99	5	− 0.29*
Easy	815	40	0.96	871	43	0.98
Very easy	1146	57	3.01	1044	52	3.23

The total count, percentage and mean person ability for the four rating scale categories are shown for two items, Item 1 as example for step ordering and Item 2 as example for step disordering

* Disordered step

## Discussion

This study aimed to validate the German version of the HLS-COVID-Q22 using Rasch analysis. The questionnaire assesses COVID-19 HL of school principals and has been developed based on the HLS-EU-Q47 [10, 40]. The HLS-EU-Q47 and its several short forms have been validated with classical test theory as well as item response theory [11, 13, 15]. Rasch analysis has been chosen, inter alia, as this analysis transforms the categorical responses of the items of the HLS-COVID-Q22 into linear measures. The analysis indicated a reliable questionnaire for the target group. Evidence was gathered for construct, fit and statistical validity.

### Dimensionality

Although, Principal component analysis of the residuals suggested possible multidimensionality, further investigation of two possible diverging item sets indicated unidimensionality with a strong disattenuation correlation value of 0.866. The two possible item sets were not random items but two sets of subscales, i.e., the subscales accessing and understanding versus the subscales appraising and applying. Boone and Staver [29] argue that the measurement tool does not need to be altered when it becomes apparent that different topics, but the same dimension is measured, i.e. an intelligence test with verbal and numerical tasks. Verbal intelligence might lie on a different part of the dimension than numerical intelligence. However, the underlying latent variable, which is measured, is still intelligence. This furthermore strengthens the assumption that the four subscales accessing, understanding, appraising and applying define one dimension, COVID-19 health literacy. Therefore, unidimensionality of the measurement tool can be assumed.

The local dependency analysis of items detected three item-pairs with low dependency. The items of the pairs were from the same subscales (Items 1 and 2: “Accessing”, Items 11 and 12: “Understanding”, Items 19 and 20: “Applying”) and could be monitored in future studies as potential redundant items. However, no concerning dependency was found, strengthening the assumption of unidimensionality.

### Item fit

Generally, the items of the HLS-COVID-Q22 showed good item fit. When evaluating different fit indices as well as reliability indices, there was no indication to delete an item. Item 8 showed Infit and Outfit MNSQ values above 1.5, but under 2.0, which implies underfit but is not degrading for the measurement tool [45]. Deleting Item 8 would slightly decrease reliability indices, which would not be an advantage from a reliability perspective. From a validity perspective, the content of Item 8 needs to be considered: “On a scale from very easy to very difficult, how easy would you say it is to understand recommendations of authorities regarding protective measures against coronavirus infection?” [10]. In Germany, recommendations of authorities changed quickly during the pandemic, especially during the time, the study was conducted (third wave of infection in spring 2021) [50]. Additionally, authorities’ recommendations were varying in the different federal states. This could have led to confusion, which might have had an impact on the item’s fit.

Item 8 should be monitored in future studies, e.g., investigate how the item functioned in another German-speaking country or investigate how the item functioned in other languages in other countries. If Item 8 keeps to show misfit, it needs to be adjusted or deleted. Additionally, item 8 was one of the items showing rather low item discrimination. Therefore, if the HLS-COVID-Q22 needs to be shortened for future research, e.g., for time reasons, item 8 could be removed. Furthermore, items that have low discrimination, step disordering, or redundant items (if several items are of similar difficulty) could be removed. For example, item 3 could be removed based on the lowest item discrimination value and step disordering.

Even though the items were generally in the reasonable range for MNSQ values of 0.6–1.4 [45], the ZSTD values were unexpectedly high and were outside of the applied threshold ( > ± 2). However, if the MNSQ values are within the reasonable range, ZSTD values can be neglected [18]. Especially in studies with large sample sizes, this phenomenon occurs. Linacre states: “So your mean-squares tell us: ‘these data fit the Rasch model usefully’, and the Z-values tell us: ‘but not exactly’.” [51]. According to the author, the statistical power to test the null hypothesis is exceptionally high because of the large sample size and inevitably results in the rejection of the null hypothesis of exact model-fit. Since the current study had a large analyzed sample size of *n* = 2.031, it can be derived that the data fit the Rasch model not exactly, but usefully. In this context, we would recommend to use the HLS-COVID-Q22 when evidence is gathered on population level as the current results suggests that the questionnaire differentiates excellent between four COVID-19 HL levels. For individual diagnostics, we would recommend to verify the psychometric properties of the HLS-COVID-Q22 in a smaller sample size of a representative German adult population before application.

The DIF analysis detected no DIF for age and sex. However, Item 13 (DIF contrast = − 0.63, *p* < .001) nearly reached the threshold of 0.64 for DIF contrast of a moderate to large effect for the sex analysis. Therefore, Item 13 should be monitored in future studies to investigate whether the item performs substantially different for females and males. Overall, evidence for fit validity, that the data fit the Rasch model for the intended purpose, was found [29, 30].

### Reliability

With a person separation index of 3.41 and person reliability of 0.92, the HLS-COVID-Q22 differentiates excellent between four COVID-19 HL levels. As it is common practice for HL questionnaires to differentiate between three or four HL levels, the developers of the HLS-COVID-Q22 recommend to classify respondents into three COVID-19 HL levels [10]. Therefore, statistical validity, that the questionnaire distinguishes between high and low COVID-19 HL levels with sufficient statistical certainty, can be assumed [29, 30]. Additionally, the values for item separation of 20.08 and item reliability of 1.0 indicate evidence for good construct validity.

### Rating scale

The 4-point likert-type rating scale worked as intended, every category was used by participants. Most items showed step ordering, except for items 2, 3, 7, 9 and 12, which exhibited step disordering. All of those five items revealed no increase but a decrease in mean person measure from rating category 1 (“very difficult”) to rating category 2 (“difficult”). However, the data points were relatively few (0–1% of all responses to a specific item) to estimate a mean person measure for rating category 1 which probably led to a degraded estimation [18]. The reason for the low usage of rating category 1 (“very difficult”) might be the target group. School principals represent a population that is well-educated and has a high income, which are known to be relevant determinants of health literacy [52]. They usually report higher HL level than a general German adult population [53, 54]. Nonetheless, the step ordering of these five items should be monitored in future studies. If rating category 1 (“very difficult”) keeps on having a low usage rate, collapsing category 1 and 2 into one category needs to be considered. In this case, having two distinct categories instead of one joint category would not help to differentiate between respondents’ ability levels [18].

### Previous findings

The present findings, indicating that the HLS-COVID-Q22 seems to be a reliable measurement tool, are in line with findings of other validation studies of the questionnaire. Reliability was investigated using mainly Cronbach’s Alpha. Here, high values have been found in all four subscales (accessing, understanding, appraising and applying) in German, Iranian and Turkish samples of adult populations with values of > 0.80, > 0.80 and > 0.90, respectively [10, 22, 23]. In the Iranian study, McDonald’s Omega values of > 0.80 replicated the findings for Cronbach’s Alpha [22]. In the Turkish study, test-retest reliability was confirmed with a test-retest reliability value greater than 0.90 and an intraclass correlation coefficient value of 0.967 [23].

To investigate dimensionality in these studies, CFA (as well as exploratory factor analysis in the Iranian study) was used to confirm a 4 factor model fit (4 subscales), showing moderate to adequate fit [10, 22, 23]. For the German HLS-COVID-Q22 only moderate fit was found after modification of the model [10]. The authors discuss that the high correlations that they have found between the subscales conflict with the assumption of four independent factors and indicate a second-order common factor. This can be confirmed by the Turkish study, that also computed a second-order factor model (with COVID-19 HL as the higher level factor) which revealed to show a better model fit than the four factor model [23]. Recently, a unidimensional model has been assumed and confirmed for an adapted version of the original HLS-EU-Q47, the HLS-Q12 [16]. The assumptions of the higher level factor of COVID-19 HL are further supported by high Cronbach’s Alpha values of 0.94 in the German and of 0.976 in the Turkish study for the overall HLS-COVID-Q22 scale [10, 23]. Linacre states that unidimensionality is a choice based on the purpose of the measurement tool [44]. Based on the theoretical construct of COVID-19 HL as well as the results from PCAR, disattenuation correlation and local dependency analysis of items, we would like to argue that the HLS-COVID-Q22 is unidimensional. The HLS-COVID-Q22 measures COVID-19 HL and the four subscales represent different topics along the same dimension. This provides further evidence on construct validity [29].

For the Turkish HLS-COVID-Q22, evidence was additionally gathered for content validity and convergent validity, e.g., high correlation between the score of the HLS-COVID-Q22 and the score of a questionnaire measuring general HL [23]. For the Persian HLS-COVID-Q22, further evidence on face and content validity was obtained in the translation and cultural adaptation process [22].

### Strengths and limitations

To the authors’ knowledge, this is the first study using Rasch analysis to evaluate the psychometric properties of the HLS-COVID-Q22. The additional evidence gathered for validity and reliability has been the computation of values for item difficulty (sample-independent item measures) as well as person ability (item-independent person measures) on one dimension (COVID-19 HL), the calculation of four reliability indices, item fit statistics, i.e., item infit and outfit, item discrimination, differential item functioning, as well as insights on rating scale functioning, i.e., rating category usage and rating step ordering [18, 29].

The HLS-COVID-Q22 is a non-universal scale and was developed in the context of the COVID-19 pandemic [10]. Fortunately, the immediate threat of the COVID-19 pandemic is over. However, the insight on the psychometric properties of the HLS-COVID-Q22 is still significant for today’s society. The HLS-COVID-Q22 is based on the HLS-EU-Q47 concept, which is a widely used instrument in HL research [10, 11]. Several HL questionnaires for different populations in different languages and countries were also developed based on the HLS-EU-Q47 concept [16, 26, 55–57]. Therefore, the current findings might be helpful for these questionnaires, e.g., the findings on rating category usage and rating step ordering. Unfortunately, the occurrence of a future pandemic is likely [58, 59]. In the case of another pandemic, the HLS-COVID-Q22 could be used as a blueprint and adapted for the specific needs. In terms of pandemic preparedness, the collection of data on school principals’ COVID-19 HL levels are vital to inform public health practitioners and politics on school principals’ needs and possible measures, e.g., tailored HL interventions. HL could be a resilience factor for future crises [38].

The convenience sample is not fully representative for all school principals and school management team members as the survey took place in only four German federal states. In Germany, the federal states are responsible for education (GG, 1949, § 30) [60]. Due to missing public statistics, no data are available on the basic population of German school principals. We would like to advocate the need for public statistics on the basic population of German school principals to facilitate representative sampling in future research. If applicable in other countries, we suggest to consult the official public data to come close to the distribution of school principals’ characteristics (age, sex, school type, urban-rural divide). This might enhance sample representativeness in future studies. Although the validation of the HLS-COVID-Q22 should be independent of the sample when Rasch analysis is applied [18], the current sample is highly specific and homogenous and non-transferable to a general adult population. Therefore, future studies are needed with a representative sample of the German adult population to replicate the findings. The current findings are specific to the German version of the HLS-COVID-Q22. Future studies are needed to evaluate the psychometric properties of translated versions of the questionnaire. Additionally, other types of validity (e.g., divergent, convergent) were not tested in the current study, which limits the interpretation of validity. Also, a self-report bias cannot be excluded for the present questionnaire, that is, using a self-report rating scale might have affected the validity of the measurement tool [61].

Furthermore, the questionnaire was only validated using Rasch analysis. The development of the HLS-COVID-Q22 with Rasch analysis would have been beneficial, because measurement tool performance could have been investigated with a test sample before the start of the survey [18]. Especially, item statistics could have been monitored from the beginning. This would have facilitated the inclusion and exclusion of items to increase reliability indices.

The concept of HL is complex and several definitions exist [8]. Therefore, different approaches (self-report questionnaires, objective performance-based tools) to measure HL are used, operationalizing different aspects of HL and thus, complicating the comparison of measurement tools [62, 63]. Self-report and performance-based HL tools often correlate low, indicating to measure different aspects of HL [64–66]. In the current study, a self-report questionnaire based on the widely used HLS-EU-Q47 questionnaire concept was applied [10–15]. There are ongoing debates whether to use self-report questionnaires in HL research as there is some criticism about the accuracy of self-report tools [67–69]. Whereas one study shows no difference in the relationship between results of performance-based and self-report HL measures and the actual outcome related to HL [70], other studies suggest that performance-based tools are better suited in predicting health-related outcomes than self-report tools [64, 71]. A recent meta-analysis suggests that both approaches might differ in their prediction accuracy based on the outcome characteristics [72]. The authors assume that self-report measures might be better for ‘soft’ outcomes like self-care behavior and performance-based measures might be better for ‘hard’ outcomes like blood-sugar levels.

The current evidence is scarce and more studies are needed to address the issue on the relationship between self-report and performance-based measures and outcome measures related to HL. Different criterions should be considered when conducting a study. For example, performance-based tools might cause discomfort for people with low literacy as they might feel ashamed by their abilities [73]. A combination of both methods might improve the measurement of the complex concept of HL which has already been applied in some studies [66, 74].

### Future research

Future validation studies are needed with a representative sample of the German adult population as well as different target populations to replicate the current findings in a sample of school principals. As already mentioned, future studies are needed, for example, to investigate if Item 8 keeps showing misfit or if step disordering still occurs if more people choose rating category 1. Additional methods could be used to further investigate the validity of the German version of the HLS-COVID-Q22, such as testing for convergent and divergent validity with related concepts (e.g., health literacy, mental health literacy) and performance-based tools. Further evidence on construct validity is needed, i.e., that the ordering and spacing of items matches the prediction from theory [29]. Further evidence on reliability could be assessed by testing the test-retest or parallel forms reliability. Although the HLS-COVID-Q22 has been validated using CTT in other languages [22, 23], the additional validation using Rasch analysis would be an asset to see if the measurement tool works differently in various languages. Especially, the exploration of item fit statistics (e.g., differential item functioning or item infit and outfit) might provide valuable insight into cultural differences in HL.

## Conclusions

The HLS-COVID-Q22 meets the requirements of a measurement tool by definition of Thurstone [31]. It appears to be suited to measure COVID-19 health literacy in school principals and members of the school management team of the 4 federal states in Germany. Through Rasch analysis, it revealed to be a reliable measurement tool within this population. Evidence for construct, statistical and fit validity was obtained. However, in light of missing evidence on other types of validity, interpretation on validity should be made cautiously. Future studies are needed to confirm and extend the current findings in a general German adult population and to monitor the functioning of items and the rating scale. The HLS-COVID-Q22 has been translated into several languages and is used globally. Therefore, the scale would benefit from a multinational validation study using Rasch analysis.

## Data Availability

The dataset generated and analyzed during the current study is not publicly available as data analyses has not been finished. When data analyses are completed, the dataset will be made publicly available. Until then, the data are available from the corresponding author upon request.

## Abbreviations

CFA: Confirmatory factor analysis
COVID-HL Network: Covid-19 Health Literacy Network
COVID-HL school survey: COVID-19 Health Literacy School Principals Survey
CTT: Classical test theory
DIF: Differential item functioning
GLOBHL: Global Health Literacy Research Network
HL: Health literacy
HLS-EU-Q47: European Health Literacy Survey Questionnaire
IRT: Item response theory
MNSQ: Mean square standardized residual
PCAR: Principal component analysis of the residuals
S.E.: Standard error
ZSTD: Standardized Z value
